# Predicting Mortality in Sepsis: The Role of Dynamic Biomarker Changes and Clinical Scores—A Retrospective Cohort Study

**DOI:** 10.3390/diagnostics14171973

**Published:** 2024-09-06

**Authors:** Norberth-Istvan Varga, Adela-Teodora Benea, Madalina-Ianca Suba, Adrian Vasile Bota, Cecilia Roberta Avram, Casiana Boru, Tiberiu Liviu Dragomir, Mirandolina Prisca, Tanasescu Sonia, Monica Susan, Florin George Horhat

**Affiliations:** 1 Doctoral School, Department of General Medicine, “Victor Babes” University of Medicine and Pharmacy, 300041 Timisoara, Romania; norberth.varga@umft.ro (N.-I.V.); adela.benea@umft.ro (A.-T.B.); madalina.suba@umft.ro (M.-I.S.); 2 Infectious Diseases and Pneumophthisiology Hospital Timisoara, 300310 Timisoara, Romania; 3 Multidisciplinary Doctoral School, “Vasile Goldis” Western University, 310419 Arad, Romania; bota.adrian-vasile@student.uvvg.ro; 4 Department of Residential Training and Post-University Courses, “Vasile Goldis” Western University, 310414 Arad, Romania; avram.cecilia@uvvg.ro; 5 Department of Medicine, “Vasile Goldis” University of Medicine and Pharmacy, 310414 Arad, Romania; boru.casiana@uvvg.ro; 6 Medical Semiology II Discipline, Internal Medicine Department, “Victor Babes” University of Medicine and Pharmacy, Eftimie Murgu Sq. No. 2, 300041 Timisoara, Romania; dragomir.tiberiu@umft.ro; 7 Department of Infectious Diseases, Faculty of Medicine, “Vasile Goldis” Western University, 310414 Arad, Romania; 8 Department of Pediatrics, “Victor Babes” University of Medicine and Pharmacy, Eftimie Murgu Sq. No. 2, 300041 Timisoara, Romania; tanasescu.sonia@umft.ro; 9 Centre for Preventive Medicine, Department of Internal Medicine, “Victor Babes” University of Medicine and Pharmacy, Eftimie Murgu Sq. No. 2, 300041 Timisoara, Romania; 10 Multidisciplinary Research Center on Antimicrobial Resistance (MULTI-REZ), Microbiology Department, “Victor Babes” University of Medicine and Pharmacy, 300041 Timisoara, Romania; horhat.florin@umft.ro

**Keywords:** sepsis, prognosis, biomarkers, procalcitonin, lactate, CRP

## Abstract

Background: The prognostic value of baseline inflammatory markers in sepsis remains controversial, with conflicting evidence regarding their association with mortality. The dynamic changes in these markers over time might offer additional insights into disease progression and patient outcomes. Methods: This retrospective observational study included 138 patients with severe infections. The inflammatory biomarkers procalcitonin (PCT), C-reactive protein (CRP), and lactate (LAC) were measured at three time points: upon hospital admission (baseline), approximately 24–48 h after admission (second measurement; M2), and 48–72 h after admission (third measurement; M3). The primary outcome was 30-day mortality. A Mann–Whitney U test was used to compare the biomarker levels between the survivors and non-survivors. A Spearman’s correlation was used to assess the relationships between the baseline parameters. A logistic regression and a receiver operating characteristic (ROC) curve analysis were employed to evaluate the prognostic value of the baseline markers and their dynamic changes. Results: The baseline LAC and SOFA score were significantly associated with 30-day mortality. The percentage decrease in PCT, CRP, and LAC from the baseline to M3 emerged as strong predictors of survival, with the ROC curve analysis demonstrating superior discriminatory ability compared to the baseline values. CRP_Delta exhibited the highest AUC (0.903), followed by PCT_Delta (0.843) and LAC_Delta (0.703). Conclusions: The dynamic changes in these inflammatory biomarkers, particularly PCT, CRP, and LAC, offer valuable prognostic information beyond their baseline levels in predicting 30-day mortality in severe infections. These findings highlight the importance of monitoring biomarker trends for early risk stratification and potential treatment guidance.

## 1. Introduction

Sepsis, a life-threatening condition arising from a dysregulated host response to infection, poses a significant global health challenge. Accounting for the estimated 49 million cases and 11 million deaths each year [1], sepsis represents a staggering 20% of all global fatalities. While various pathogens can trigger sepsis, bacterial infections, which are often complicated by antibiotic resistance and toxin production, remain the primary culprits. The effective management of bacterial sepsis demands a multi-pronged approach, encompassing timely antibiotic therapy, fluid resuscitation, and swift identification and control of the infection source [2].

The pathophysiology of sepsis involves a complex interplay of both pro-inflammatory and anti-inflammatory responses, which are triggered by an invading pathogen [3]. The dysregulation of these responses leads to the release of various inflammatory markers into the bloodstream, including procalcitonin (PCT) [4], lactate (LAC) [5], and C-reactive protein (CRP) [6]. PCT, a precursor of the hormone calcitonin, is primarily produced in the thyroid gland but is also released by various other tissues in response to bacterial infections. The rise in PCT levels during sepsis is attributed to the stimulation of its production by bacterial endotoxins and pro-inflammatory cytokines. LAC, a byproduct of anaerobic metabolism, accumulates when tissue oxygen delivery is impaired, which is a hallmark of sepsis-induced hypoperfusion and organ dysfunction [7]. The elevation of CRP, an acute-phase protein synthesized by the liver, is triggered by pro-inflammatory cytokines, reflecting the systemic inflammatory response in sepsis [8].

The measurement of these biomarkers might hold significant potential as prognostic indicators in sepsis, as these factors are critical determinants of disease severity and outcome [9,10,11,12,13,14,15]. Furthermore, the true power of these biomarkers may lie not just in their initial values, but in their dynamic changes over time. Tracking these fluctuations might offer a more nuanced understanding of the patient’s clinical trajectory, distinguishing those responding favorably to therapy from those at risk of deterioration.

Driven by this potential, our study aimed to investigate the prognostic value of PCT, CRP, and LAC in patients with severe infections. We sought to determine whether their baseline levels, as well as their dynamic changes over the initial 72 h of their hospitalization, could predict 30-day mortality. Additionally, we explored the inter-relationships between these biomarkers and other clinical parameters at the baseline, aiming to enhance our understanding of their combined roles in risk stratification and treatment guidance. Our choice of these specific biomarkers was motivated by their widespread availability, relatively low cost, and extensive study in the context of sepsis, making them accessible and clinically relevant tools for prognostication in diverse healthcare settings worldwide.

## 2. Materials and Methods

### 2.1. Study Design

This investigation employed an observational retrospective cohort study design. Patient data were collected from individuals admitted to the Infectious Disease Clinic of the Infectious Disease Hospital in Timisoara, a tertiary teaching hospital, between July 2023 and June 2024. The study population consisted of 138 adult patients (aged > 18 years) who fulfilled the Sepsis-3 criteria as defined by Singer et al. in 2015. These patients were admitted to our unit directly from the Emergency Department or general medical wards within approximately 24 h of suspected sepsis onset. The diagnostic criteria for sepsis included the presence of a suspected or proven infection coupled with an acute increase in SOFA score of ≥2 points. Exclusion criteria included age under 18 years, post-trauma or post-surgery status, burn injuries, current malignancy, and immunosuppression (e.g., due to therapy or cancer). Patients who did not survive to the second or third measurement point were also excluded. The study protocol was reviewed and approved by the hospital’s ethics committee, and all procedures were performed in accordance with the Declaration of Helsinki. Informed consent was obtained from all participants or their legal representatives upon admission.

### 2.2. Data Collection

For each enrolled patient, a comprehensive dataset was compiled, encompassing patient demographics (age, gender), vital signs at admission (pulse, blood pressure, oxygen saturation, etc.), and a detailed medical history to ascertain comorbidities. The Charlson Comorbidity Index (CCI) was subsequently calculated based on the documented comorbidities. The initial Sequential Organ Failure Assessment (SOFA) score was recorded upon admission, reflecting the severity of organ dysfunction. A broad spectrum of biochemical parameters were assessed, including white blood cell count (WBC), neutrophils, lymphocytes, creatinine, bilirubin, glucose, albumin, platelet count, etc. The inflammatory biomarkers procalcitonin (PCT), C-reactive protein (CRP), and lactate (LAC) were measured at three time points: upon hospital admission (baseline), approximately 24–48 h after admission (second measurement; M2), and 48–72 h after admission (third measurement; M3). The percentage changes in PCT, CRP, and LAC from baseline to the third measurement (PCT_Delta, CRP_Delta, and LAC_Delta, respectively) were calculated as follows: ((M3 Value − Baseline Value)/Baseline Value) * 100. The source of infection, as determined during the course of clinical care, was also documented. The primary outcome of interest was 30-day mortality, assessed through follow-up at 30 days post-admission, with outcome coded as 0 for survival (S) and 1 for non-survival (NS).

Procalcitonin levels were measured using the Roche Elecsys BRAHMS PCT assay (Roche Diagnostics, Mannheim, Germany) (measuring range: 0.02 to 100 ng/mL). C-reactive protein was assessed with the Roche Cobas Integra system (measuring range: 0.05 to 350 mg/L). Lactate measurements were obtained using point-of-care lactate meters (measuring range: 0.5 to 20 mmol/L). All measurements were conducted in accordance with standard laboratory protocols.

### 2.3. Statistical Analysis

The normality of continuous variables was assessed using the Kolmogorov–Smirnov test. For variables exhibiting a normal distribution, parametric tests were employed. However, due to the prevalent non-normal distribution observed in many clinical and laboratory parameters, non-parametric tests were utilized for the majority of analyses. The Mann–Whitney U test was used to compare differences between survivors and non-survivors. Spearman’s rank correlation coefficient was calculated to assess the relationships between baseline clinical and laboratory parameters. Logistic regression analysis was performed to evaluate the association between these parameters and 30-day mortality, both at baseline and when considering their dynamic changes over time. The discriminatory ability of different models was assessed using receiver operating characteristic (ROC) curve analysis, with the area under the curve (AUC) serving as the primary metric for comparison. Statistical significance was set at *p* < 0.05. All analyses were conducted using SPSS version 26.

## 3. Results

### 3.1. Overall Characteristics at Baseline

The study cohort consisted of 138 patients with sepsis, of which 63 (45.7%) were female and 75 (54.3%) were male. The median age was 75 years (IQR 69–82), with the non-survivors being significantly older than the survivors (median of 79 vs. 72 years, *p* < 0.05). The median duration of hospital stay was 10 days (IQR 7–12), with the non-survivors having a slightly longer stay compared to the survivors (median of 10 vs. 7 days). The median SOFA score at the baseline was 5 (IQR of 4–7), with the non-survivors exhibiting a higher median score of 6 compared to 4 in the survivors (*p* < 0.05).

The most common infection site was respiratory (42.8%), followed by genitourinary (23.2%). The median Charlson Comorbidity Index (CCI) was 6 (IQR of 4–7), with no significant difference between the survivors and non-survivors. Hypertension and diabetes were the most prevalent comorbidities, affecting 68.1% and 77.5% of the patients, respectively.

Regarding the baseline laboratory findings, the non-survivors displayed significantly higher lactate levels (median 3.37 mmol/L vs. 2.71 mmol/L, *p* < 0.05) and a higher Neutrophil-to-Lymphocyte Ratio (NLR) (median 9.4 vs. 7.58, *p* < 0.05) compared to the survivors. No significant differences were observed between the two groups in terms of their baseline procalcitonin (PCT), C-reactive protein (CRP), white blood cell count (WBC), creatinine, total bilirubin, platelet count, albumin, glucose, or mean blood pressure. The overall characteristics at baseline are summarized in Table 1. 

### 3.2. Dynamic Changes in Plasma PCT, CRP, and LAC

The results of this study indicate that larger decreases in the levels of PCT, CRP, and LAC over the first 72 h are associated with improved survival in sepsis patients. Specifically, the median decrease in PCT level was more pronounced in the survivors (−45.02%) compared to the non-survivors (−17.24%). Similarly, the survivors exhibited a greater median decrease in their CRP level (−45.71%) than the non-survivors (−30.83%). Although less pronounced, a trend towards larger decreases in lactate levels was also observed in the survivors (−34.02%) compared to the non-survivors (−26.91%). These findings are summarized in Table 2.

### 3.3. Normality of Distribution and Kolmogorov–Smirnov Test

The Kolmogorov–Smirnov test was employed to assess the normality of distribution for the various clinical and laboratory parameters. The results indicate that only the CRP_Delta followed a normal distribution (*p* > 0.05). In contrast, the remaining variables—age, WBC, NLR, PCT, CRP, and LAC, at various time points, and the CCI and SOFA scores—exhibited significant deviations from normality (*p* < 0.05).

These findings highlight the non-normal distribution that was prevalent in many of our clinical and laboratory parameters. In order to maintain the robustness of our statistical analyses and ensure accurate inferences, we employed non-parametric methods when analyzing these variables.

The Kolmogorov–Smirnov test results for the normality assessment are available in the Supplementary Materials (Table S1).

### 3.4. Comparison of Baseline Characteristics and Biomarker Dynamics between Survivors and Non-Survivors

The Mann–Whitney U test was employed to compare the distributions of the various variables between the survivor (S) and non-survivor (NS) groups. The Mann–Whitney U test results revealed several significant differences between the survivors and non-survivors across the various clinical and laboratory parameters.

Significant differences (*p* < 0.05) were observed for age, CCI, NLR, PCT_Delta, CRP_Delta, LAC_Base, LAC_Delta, and SOFA_Base, suggesting their potential value in predicting the outcome of sepsis.

In contrast, the WBC, PCT_Base, and CRP_Base did not show statistically significant differences between the two groups. Notably, the significant *p*-values for the PCT_Delta and CRP_Delta underscored the strong association between the changes in these biomarkers and the outcome. The lack of significance for the WBC, PCT_Base, and CRP_Base indicated that their baseline values may not be strong predictors of the outcome in this study. These findings highlight the importance of considering the dynamics of biomarker changes, rather than just their baseline values, when assessing their prognostic significance.

The Mann–Whitney U test results are available in the Supplementary Materials (Table S2).

### 3.5. Correlations between Baseline Biomarkers

The Spearman’s correlation analysis was performed to evaluate the associations between the baseline clinical and laboratory parameters. As expected, a strong positive correlation was observed between age and the Charlson Comorbidity Index (CCI) (rho = 0.471, *p* < 0.001), reflecting the age-dependent nature of the CCI. Additionally, the baseline PCT and CRP levels demonstrated a moderate positive correlation (rho = 0.451, *p* < 0.001), suggesting a degree of concordance in their inflammatory response. The CRP levels also showed a moderate positive correlation with the baseline LAC (rho = 0.551, *p* < 0.001).

Furthermore, the SOFA_Base exhibited weak positive correlations with both age (rho = 0.261, *p* = 0.002) and LAC_Base (rho = 0.177, *p* = 0.038), hinting at the potential influence of age and inflammation on the baseline organ dysfunction. The remaining correlations were either weak or not statistically significant. These findings provide insights into the inter-relationships between various baseline parameters, which can inform further analyses exploring their combined impacts on patient outcomes.

The Spearman’s rank correlation coefficients for the baseline clinical and laboratory parameters are detailed in the Supplementary Materials (Table S3).

### 3.6. Logistic Regression

To investigate the association between the baseline clinical and laboratory parameters and 30-day mortality, we employed a logistic regression analysis, adjusting for potential confounders. Our findings revealed that the Charlson Comorbidity Index (CCI), Neutrophil-to-Lymphocyte Ratio (NLR), baseline lactate level (LAC_Base), and baseline SOFA score (SOFA_Base) were significant predictors of mortality. Notably, for each one-unit increase in the CCI, the odds of mortality increased by 31.6%, while a one-unit increase in the NLR was associated with a 47% increase in the odds of mortality. The baseline lactate level emerged as a particularly strong predictor, with each one-unit increase more than doubling the odds of mortality. Similarly, the baseline SOFA score demonstrated a substantial impact, with each one-unit increase leading to a 73.5% increase in the odds of mortality.

In contrast, age, white blood cell count (WBC), baseline PCT, and baseline CRP did not exhibit significant associations with 30-day mortality in this model. These results underscore the importance of considering both clinical parameters and specific inflammatory markers, particularly lactate and NLR, when assessing the risk of mortality in this patient population. However, it is crucial to acknowledge that this analysis focused solely on the baseline values. Future investigations will incorporate the dynamic changes in biomarkers over time to gain a more comprehensive understanding of their prognostic significance and potential for early risk stratification. Table S4 in the Supplementary Materials shows the correlation between the baseline variables and the outcome.

To investigate the prognostic value of the changes in the inflammatory markers, we calculated the percentage decrease in the PCT, CRP, and LAC levels, from baseline to M3, and included these in a logistic regression model alongside the baseline clinical parameters. This analysis revealed that decreases in the PCT, CRP, and LAC levels were significantly associated with a reduced 30-day mortality. Specifically, for each 1% decrease in the PCT, CRP, and LAC levels, from baseline to M3, the odds of 30-day mortality decreased. This is reflected in the odds ratios of 0.948, 0.762, and 0.887, respectively. To illustrate this, the odds ratio of 0.762 for the CRP_Delta signifies that for every 1% decrease in CRP levels over the first three days, the odds of mortality are reduced by approximately 24% (1 − 0.762 = 0.238), holding other factors constant. Similarly, a 1% decrease in the PCT level corresponds to a 5.2% reduction in mortality odds (1 − 0.948 = 0.052), and a 1% decrease in the LAC level translates to an 11.3% reduction in mortality odds (1 − 0.887 = 0.113). These findings underscore the critical importance of monitoring the dynamics of these biomarkers, as their decline over time emerges as a potent indicator of improved survival. It is important to note that the biomarker delta values (PCT_Delta, CRP_Delta, LAC_Delta) were calculated as a percentage decrease from the baseline to M3. This transformation was deliberately performed to ensure that a higher delta value represents a greater reduction in the biomarker level, aligning with the intuitive interpretation that a decrease in inflammatory markers is associated with improved outcomes. Table S5 in the Supplementary Materials offers the details of the correlation between the dynamic changes of the variables and outcome.

### 3.7. ROC Curves

**Figure 1:**
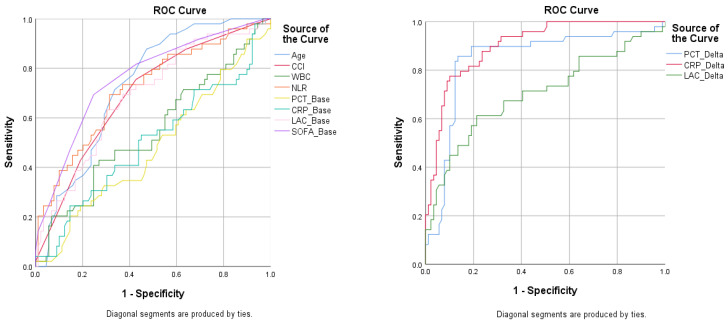
ROC curves of the baseline variables and their corresponding percentage of change within the 72 h timeframe (third measurement).

The ROC curve analysis further underscored the superior prognostic value of biomarker dynamics in predicting 30-day mortality. In order to visualize the differences between the ROC curves better, we performed the tests for the baseline values of biomarkers and their corresponding percentage of change separately. The AUCs for the percentage decreases in the PCT, CRP, and LAC levels, from baseline to M3 (PCT_Delta: 0.843, CRP_Delta: 0.903, and LAC_Delta: 0.703), were notably higher than those for their corresponding baseline values (PCT_Base: 0.478, CRP_Base: 0.540, and LAC_Base: 0.689). This signifies that observing the changes in these biomarkers over time provides a more accurate assessment of a patient’s risk of mortality compared to relying solely on their initial levels. Among the baseline predictors, the SOFA score exhibited the highest discriminatory power (AUC = 0.762), followed by age (0.730) and NLR (0.716), suggesting a moderate ability to distinguish between the survivors and non-survivors. In contrast, the CCI (0.609) and WBC (0.546) demonstrated weaker predictive capabilities, with the WBC approaching the level of a random classifier (AUC = 0.5). These findings emphasize the potential clinical utility of incorporating biomarker trends into prognostic models for patients with severe infections. The ROC curves for baseline variables and their corresponding percentage of change are presented in Figure 1. The area under the ROC curve for baseline values and their corresponding percentage of change is shown in Table 3. 

## 4. Discussion

### 4.1. Baseline Values

The relationship between baseline inflammatory markers and mortality in severe infections has been extensively investigated, yet the findings remain heterogeneous and are often contradictory. Our study, which found no significant association between the baseline PCT and CRP levels and 30-day mortality, aligns with several previous reports. For instance, both Poddar et al. [16] and Claeys et al. [17] observed no difference in the baseline PCT levels between the survivors and non-survivors in their respective cohorts. Similarly, Durrance [18] reported that markedly elevated baseline PCT levels did not correlate with mortality in their study population. The lack of association between the baseline WBC count and mortality in our study is also consistent with the findings of Claeys et al. [17]. These studies collectively suggest that the prognostic value of baseline PCT, CRP, and WBC might be limited, at least in certain patient populations or clinical settings.

However, other studies have presented contrasting results, highlighting the complexity of this issue. For example, Giamarellos-Bourboulis et al. [19] and Jain et al. [20] reported that elevated baseline PCT levels were indeed associated with increased mortality risk. Moreover, Li et al. [21] found that baseline PCT, along with sTREM-1, were independent predictors of 28-day mortality. The discrepancy between these findings and our own could be attributed to variations in study design, patient populations, severity of illness, or the specific assays used for biomarker measurement. The heterogeneity in the literature underscores the need for further research to elucidate the complex relationship between baseline inflammatory markers and patient outcomes in sepsis.

In our study, we observed a notable difference in the Neutrophil-to-Lymphocyte Ratio (NLR) between the survivors and non-survivors. The median NLR was significantly higher in the non-survivors (9.4) compared to survivors (7.58). This observation aligns with previous research highlighting the importance of the Neutrophil-to-Lymphocyte Ratio in assessing both severity and risk in infections, where an elevated NLR has been associated with an increased mortality risk [22]. These results are echoed by Ljungström et al. (2017), who demonstrated the effectiveness of the NLR, particularly in combination with other biomarkers, for diagnosing bacterial sepsis and predicting severe outcomes [23].

Overall, our results contribute to the ongoing discussion regarding the prognostic value of baseline inflammatory markers in severe infections. While certain markers like lactate levels and SOFA score appear to consistently predict mortality, the role of PCT and CRP levels at the baseline remains less clear and might be influenced by various factors. The heterogeneity in the literature underscores the need for further research to elucidate the complex interplay between these biomarkers and patient outcomes.

### 4.2. Biomarker Kinetics

The superior prognostic value of biomarker kinetics, particularly the association between a decrease in inflammatory markers and improved outcomes, resonates with several studies in the literature. Our observation that a reduction in PCT levels over time is associated with better survival aligns with the findings of Poddar et al. [16], Schuetz et al. [24,25], and Pieralli et al. [11]. These studies, like ours, highlight the importance of monitoring PCT kinetics, with some even proposing specific percentage decreases (e.g., >80% or >50%) as potential thresholds for predicting survival. Similarly, the association between a CRP reduction and favorable outcomes echoes the observations of Bahloul et al. [26] and Rios-Toro et al. [27], who also reported a correlation between decreasing CRP levels and improved prognosis. The significance of lactate clearance in predicting survival, as observed in our study, is further supported by the work of Nguyen et al. [12], who found that early lactate clearance was associated with decreased mortality in severe sepsis and septic shock. The convergence of these findings strengthens the evidence supporting the use of biomarker kinetics as a valuable tool for prognostication in sepsis.

While the majority of studies emphasize the importance of biomarker dynamics, some have focused primarily on baseline values or have reported conflicting results. For instance, Charles et al. [28] found that the initial PCT level failed to predict the outcome, although the PCT kinetics between days 2 and 3 were significantly associated with survival. Similarly, Claeys et al. [17] observed that the inaugural PCT and CRP levels poorly predicted the outcome, but decreasing levels were associated with a higher probability of survival. These discrepancies might stem from differences in the study populations, timing of biomarker measurements, or specific statistical methods employed.

### 4.3. Biomarker Correlations at Baseline

The assessment of the correlations between the baseline biomarkers in our study revealed a moderate positive correlation between the PCT and CRP levels (rho = 0.451, *p* < 0.001), as well as between the CRP and LAC levels (rho = 0.551, *p* < 0.001). These findings suggest a degree of concordance in the inflammatory response, reflected by these markers at the time of admission. The observed correlation between the PCT and CRP levels aligns with previous studies that have reported similar associations. For instance, Claeys et al. [17] also noted a correlation between the initial PCT levels and CRP values in patients with septic shock.

However, the literature on biomarker correlations in sepsis is not entirely consistent. Some studies have reported correlations between PCT levels and other markers like IL-6 [21], while others have found no significant associations between PCT and various cytokines (Oberholzer et al. [29]). The variability in these reported correlations might be attributed to differences in the study populations, disease severity, or timepoints of biomarker measurement. The moderate correlations observed in our study contribute to this ongoing discussion and suggest that while PCT, CRP, and LAC levels may exhibit some degree of interdependence at the baseline, their relationship is not necessarily strong or universally consistent across different patient cohorts.

### 4.4. Strengths and Limitations

The present study benefits from its comprehensive assessment of both baseline values and dynamic changes in inflammatory biomarkers, offering a nuanced understanding of their prognostic significance in severe infections. The inclusion of a diverse range of clinical and laboratory parameters, coupled with robust statistical methodology, strengthens the validity of our findings. The focus on biomarker kinetics, particularly the percentage decreases in the PCT, CRP, and LAC levels, provides insights into their potential for early risk stratification and treatment guidance. The utilization of both non-parametric tests and logistic regression with an adjustment for confounders further enhances the rigor of our analysis.

The retrospective nature of our study design inherently introduces potential limitations. The reliance on existing medical records might lead to incomplete or inconsistent data collection, potentially impacting the accuracy of our findings. The relatively small sample size, while adequate for identifying significant associations, might limit the generalizability of our results to broader populations. The single-center setting restricts the assessment of potential variations in biomarker dynamics and outcomes across different healthcare environments. Additionally, the observational nature of this study precludes definitive conclusions about causality, warranting further research to establish the mechanistic links between biomarker changes and patient outcomes. Finally, our focus on 30-day mortality as the primary outcome might not capture the full spectrum of long-term consequences associated with severe infections.

## 5. Conclusions

The present study underscores the superior prognostic value of dynamic changes in inflammatory biomarkers, particularly in PCT, CRP, and LAC values, over their baseline values in predicting 30-day mortality in patients with severe infections. The decline in these biomarkers over the initial 72 h of hospitalization emerged as a robust indicator of improved survival, independent of other clinical and laboratory parameters. These findings highlight the importance of incorporating biomarker kinetics into clinical practice for enhanced risk stratification and potentially guiding therapeutic interventions in this critical patient population.

## Figures and Tables

**Table 1:** Overall Characteristics at Baseline.

	Overall Population; Median (25th–75th Percentile)	Survivors; Median (25th–75th Percentile)	Non-Suvrivors; Median (25th–75th Percentile)
Age	75 (69–82)	72 (65–79)	79 (75–86)
Sex	Female = 63 (45.7%), Male = 75 (54.3%)		
Duration of Stay in Hospital		10 (7–12)	7 (5–11)
SOFA Score	5 (4–7)	4 (3–5)	6 (5–7)
Infection Site:			
Respiratory	59		
Genitourinary	32		
Skin and soft tissue	19		
Gastrointestinal	18		
Musculoskeletal	6		
Others	4		
CCI Score	6 (4–7)	5 (4–6)	6 (5–7)
Comorbidities:			
Hypertension	94		
Coronary Artery Disease	51		
Heart Arrhythmia	35		
Chronic Heart Failure	32		
Stroke	40		
Vascular/Alzheimer/Mixed Dementia	35		
COPD	31		
Diabetes	107		
Liver Disease	28		
Renal Disease	34		
Malignancies in history	12		
Laboratory Findings at Baseline			
Procalcinonin (ng/mL)	2.97 (0.94–10.57)	2.99 (1.03–9.82)	2.79 (0.91–10.2)
CRP (mg/L)	157 (97.9–226.57)	155 (98.5–214.98)	165 (74.5–234.8)
Lactate (mmol/L)	2.9 (2.16–3.93)	2.71 (1.99–3.68)	3.37 (2.64–4.95)
Creatinine (mg/dL)	2.21 (1.53–2.96)	2.16 (1.55–2.88)	2.23 (1.5–2.99)
Total Bilirubin (mg/dL)	1.47 (0.74–2.58)	1.39 (0.74–2.44)	1.51 (0.75–2.65)
Glucose (mg/dL)	133 (101–157)	130 (99–166)	134 (101–160)
Albumin (g/dL)	3.69 (3.03–4.67)	3.74 (3.23–4.91)	3.51 (3–4.61)
WBC (×1000/µL)	15.9 (12.1–21.5)	15.98 (12.19–19)	15.94 (12.28–21.24)
Platelet count (×1000/µL)	177 (130–224)	181 (131–230)	159 (127–222)
NLR	8.53 (6.48–9.94)	7.58 (6.18–9.28)	9.4 (8.2–10.96)
Mean Blood Pressure (mmHg)	88.3	88	88.5

**Table 2:** Median Values and Percentage changes of PCT, CRP, and LAC levels in Survivors and Non-survivors at Baseline and 72 Hours. M3 = plasma values at the third measurement.

Biomarker	Overall Population (*n* = 144)	Survivors (*n* = 103)	Non-Survivors (*n* = 41)	*p*-Value
PCT at baseline (median)	2.97	2.99	2.87	0.672
PCT at M3 (median)	1.67	1.57	1.94	0.245
PCT_Delta (%)	−38.41	−45.02	−17.24	0
CRP at baseline (median)	157	155	168.01	0.95
CRP at M3 (median)	92.58	83.71	101.07	0.1
CRP_Delta (%)	−41.28	−45.71	−30.83	0
LAC at baseline (median)	2.9	2.71	3.43	0
LAC at M3 (median)	1.95	1.77	2.69	0
LAC_Delta (%)	−32.17	−34.02	−26.91	0

**Table 3:** Area Under the ROC Curve for baseline values and their corresponding percentage of change.

Area Under the Curve	
Test Result Variables	Area
Age	0.730
CCI	0.694
WBC	0.546
NLR	0.716
PCT_Base	0.478
CRP_Base	0.503
LAC_Base	0.689
SOFA_Base	0.762
PCT_Delta	0.843
CRP_Delta	0.903
LAC_Delta	0.703

## Data Availability

Data is available upon request from the corresponding author.

## Supplementary Materials

The following supporting information can be downloaded at: https://www.mdpi.com/article/10.3390/diagnostics14171973/s1; Table S1: Kolmogorov–Smirnov Test for Normality of Distribution; Table S2: Mann–Whitney U test; Table S3: Spearman’s Correlation Test; Table S4: Correlation Between Baseline Variables and Outcome; Table S5: Correlation Between the Dynamic Changes of Variables and Outcome.
