# Supplementary material for: Predicting Mortality in Sepsis: The Role of Dynamic Biomarker Changes and Clinical Scores—A Retrospective Cohort Study
Source: Diagnostics (Basel). 2024 Sep 6;14(17):1973. doi: 10.3390/diagnostics14171973 (PMC11393859; doi:10.3390/diagnostics14171973)
Supplement: Supplementary file 1 [file diagnostics-14-01973-s001.zip › diagnostics-3181713-supplementary.pdf]

diagnostics

Article

Predicting Mortality in Sepsis: The Role of Dynamic Biomarker Changes and Clinical Scores – A Retrospective Cohort Study

Supplementary Material

Table S1. Kolmogorov-Smirnov Test for Normality of Distribution

	Kolmogorov-Smirnov[a]		
	Statistic	df	Sig.
Age	.114	138	.000
CCI	.149	138	.000
WBC	.078	138	.041
NLR	.093	138	.006
PCT_Base	.314	138	.000
PCT_D2	.313	138	.000
PCT_D3	.314	138	.000
PCT_Delta	.129	138	.000
CRP_Base	.088	138	.011
CRP_D2	.099	138	.002
CRP_D3	.131	138	.000
CRP_Delta	.062	138	.200*
LAC_Base	.098	138	.002
LAC_D2	.087	138	.012
LAC_D3	.084	138	.017
LAC_Delta	.096	138	.003
SOFA_Base	.171	138	.000

Table S2. Mann-Whitney U Test

Test Statistics[a]				
	Mann-Whitney U	Wilcoxon W	Z	Asymp. Sig. (2-tailed)
Age	1175.500	5180.500	-4.475	.000
CCI	1333.000	5338.000	-3.852	.000
WBC	1982.000	5987.000	-.883	.377
NLR	1239.000	5244.000	-4.189	.000

PCT_Base	2085.500	3310.500	-.423	.672
PCT_Delta	686.500	1911.500	-6.647	.000
CRP_Base	2166.500	6171.500	-.062	.950
CRP_Delta	424.500	1649.500	-7.813	.000
LAC_Base	1358.000	5363.000	-3.660	.000
LAC_Delta	1294.000	2519.000	-3.944	.000
SOFA_Base	1036.500	5041.500	-5.164	.000
Grouping Variable: Outcome				

Table S3. Spearman's Correlation Test

			Age	CCI	WBC	NLR	PCT_Base	CRP_Base	LAC_Base	SOFA_Base
Spearman's rho	Age	Correlation Coefficient	1.000	.471**	.118	.098	-.061	.057	.153	.261**
		Sig. (2-tailed)	.	.000	.168	.255	.476	.507	.073	.002
		N	138	138	138	138	138	138	138	138
	CCI	Correlation Coefficient	.471**	1.000	-.041	.222**	-.038	-.011	.126	.113
		Sig. (2-tailed)	.000	.	.631	.009	.657	.902	.141	.188
		N	138	138	138	138	138	138	138	138
	WBC	Correlation Coefficient	.118	-.041	1.000	.126	.127	.151	.099	.024
		Sig. (2-tailed)	.168	.631	.	.140	.137	.078	.247	.780
		N	138	138	138	138	138	138	138	138
	NLR	Correlation Coefficient	.098	.222**	.126	1.000	-.004	.008	.173*	.145
		Sig. (2-tailed)	.255	.009	.140	.	.961	.928	.042	.089
		N	138	138	138	138	138	138	138	138
	PCT_Base	Correlation Coefficient	-.061	-.038	.127	-.004	1.000	.451**	.254**	.067
		Sig. (2-tailed)	.476	.657	.137	.961	.	.000	.003	.433
		N	138	138	138	138	138	138	138	138
	CRP_Base	Correlation Coefficient	.057	-.011	.151	.008	.451**	1.000	.551**	.085
		Sig. (2-tailed)	.507	.902	.078	.928	.000	.	.000	.319
		N	138	138	138	138	138	138	138	138
	LAC_Base	Correlation Coefficient	.153	.126	.099	.173*	.254**	.551**	1.000	.177*
		Sig. (2-tailed)	.073	.141	.247	.042	.003	.000	.	.038
		N	138	138	138	138	138	138	138	138
	SOFA_Base	Correlation Coefficient	.261**	.113	.024	.145	.067	.085	.177*	1.000
		Sig. (2-tailed)	.002	.188	.780	.089	.433	.319	.038	.
		N	138	138	138	138	138	138	138	138

Table S4. Correlation Between Baseline Variables and Outcome

Variables in the Equation									
								95% C.I.for EXP(B)	
		B	S.E.	Wald	df	Sig.	Exp(B)	Lower	Upper
Step 1[a]	Age	.070	.030	5.421	1	.020	1.072	1.011	1.137
	CCI	.275	.195	1.981	1	.159	1.316	.898	1.930
	WBC	.027	.041	.420	1	.517	1.027	.948	1.113
	NLR	.386	.131	8.620	1	.003	1.470	1.137	1.902
	PCT_Base	-.006	.011	.323	1	.570	.994	.972	1.016
	CRP_Base	-.007	.003	4.324	1	.038	.993	.987	1.000
	LAC_Base	.720	.258	7.798	1	.005	2.053	1.239	3.403
	SOFA_Base	.551	.146	14.150	1	.000	1.735	1.302	2.312
	Constant	-15.330	2.920	27.561	1	.000	.000		

Table S5: Correlation Between the Dynamic Changes of Variables and Outcome

Variables in the Equation									
								95% C.I.for EXP(B)	
		B	S.E.	Wald	df	Sig.	Exp(B)	Lower	Upper
Step 1[a]	Age	.072	.035	4.161	1	.041	1.074	1.003	1.151
	CCI	.219	.278	.621	1	.431	1.245	.722	2.148
	SOFA_Base	.350	.189	3.439	1	.064	1.420	.980	2.056
	PCT_Delta	-.054	.015	12.206	1	.000	.948	.920	.977
	CRP_Delta	-.272	.066	17.072	1	.000	.762	.670	.867
	LAC_Delta	-.120	.043	7.788	1	.005	.887	.815	.965
	Constant	6.371	3.742	2.899	1	.089	584.596		
